# Health anxiety and the negative interpretation of children’s bodily symptoms in mothers of cancer patients

**DOI:** 10.1007/s11764-023-01431-z

**Published:** 2023-07-20

**Authors:** Parham Hosseinchi, Erfan Ghalibaf, Golnoosh Kamyab, Aziz Eghbali, Ali Khatibi

**Affiliations:** 1 Institute for Cognitive Science Studies, Tehran, Iran; 2 Department of Anatomy, Université du Québec à Trois-Rivières, Trois-Rivieres, QC Canada; 3 CogNAC Research Group, Université du Québec à Trois-Rivières, Trois-Rivieres, QC Canada; 4 Department of Neuroscience, Iran University of Medical Sciences, Tehran, Iran; 5 Ali-Asghar Clinical Research Development Center, Iran University of Medical Sciences, Tehran, Iran; 6 Centre of Precision Rehabilitation for Spinal Pain (CPR Spine), School of Sport, Exercise and Rehabilitation Sciences, University of Birmingham, Birmingham, UK; 7 Centre for Human Brain Health, University of Birmingham, Birmingham, UK; 8 Institute for Mental Health, University of Birmingham, Birmingham, UK

**Keywords:** Caregivers, Cancer, Fear of progression, Health anxiety, Interpretation bias, Quality of life

## Abstract

**Purpose:**

Fear of progression (FoP) is a substantial concern for family caregivers of cancer survivors and is related to a number of adverse outcomes, including increased mental distress and worse quality of life. Previous research has revealed that health anxiety (HA) contributes to fear of relapse, but cognitive factors underlying establishing and maintaining FoP in mothers of cancer patients have not been examined. In this study, we were looking to investigate this association.

**Methods:**

We used the computerized interpretation bias (IB) assessment to investigate the biased interpretation of ambiguous bodily information and its association with FoP through HA among 69 mothers of cancer patients and 42 mothers of healthy kids.

**Results:**

Mothers of cancer patients interpreted more negatively ambiguous bodily symptoms than mothers of healthy kids. Moreover, they had higher levels of HA and FoP and lower quality of life than the healthy group. Also, among mothers of cancer patients, the relationship between negative IB and FoP is mediated by their HA.

**Conclusions:**

The findings of this study imply that negative IB may contribute to increased HA, which in turn contributes to higher levels of FoP among the mothers of cancer patients, which may reduce the quality of life of their children.

**Implications for Cancer Survivors:**

From these findings, we propose that changing HA through modification of IB might lower the FoP in mothers of kids with cancer and improve the mother and child’s quality of life.

## Introduction

Cancer is the second leading cause of death among children aged 1 to 14. According to the American Cancer Society, about 1050 children in the USA lost their lives to cancer in 2022 [1]. The impact of cancer goes beyond the patient and affects the caregiver’s quality of life and well-being. Mothers, who often bear the brunt of the caregiving burden, face significant distress when their child is diagnosed with cancer [2]. They play a crucial role in providing emotional and mental support to their family members [3]. Moreover, studies show that mothers tend to experience more stress than fathers while caring for their children with cancer [4, 5]. Even though treatment has improved and survival rates have increased, cancer survivors still face potential long-term health and well-being challenges [6].

Illness anxiety disorder, also known as hypochondriasis or health anxiety (HA), is an excessive fear of becoming very sick [7]. Research suggests that HA may develop in childhood [8, 9]. Also, personal experience with a disease or a person who has the disease, such as family members or a close friend, is considered a vulnerability factor for developing HA [10, 11]. According to the cognitive-behavioral model, internal (such as physical symptoms) or external (such as hearing about a friend’s illness) information can increase our anxiety about our health, behavior, and decisions [12]. Studies show that when people are threatened, they are more biased by internal information than the ones they receive from the object of the threat [13].

Previous studies have suggested a possible link between anxiety-associated bias in interpreting ambiguous distress/threat-related information among children and their mothers [14, 15]. Moreover, several studies indicated that caregivers of patients who suffer from pain have a cognitive bias for pain and caregiver biases may be linked to increased pain interference and impaired pain interpretation in the patient’s life [16–18].

Fear of progression (FoP) has been identified as a reactive and conscious fear that can arise based on the experience of a chronic disease such as cancer [19, 20]. Fear of progression is a common psychological long-term effect in parents of cancer patients [21, 22]. It has been suggested that this fear goes beyond the patient and involves caregivers. Parents’ FoP were similar to adult cancer patients and their partners [23] and can lead to impaired family or role-functioning [24, 25], reduced quality of life [23, 24], and poor social, emotional, and behavioral development in the child [26, 27].

In the context of diseases with a chance of recurrence, it has been shown that biased processing of disease-related information can contribute to HA and fear of recurrence among patients [28]. Understanding the relationship between those biases in caregivers and their HA is essential since we know that the caregiver’s quality of life ultimately impacts patients’ well-being and treatment outcomes. However, no studies have investigated the relationship between HA and negative interpretation of children’s bodily symptoms in mothers of cancer patients. In this study, we predicted that mothers of cancer patients have negative interpretations of ambiguous situations, which can increase their FoP through HA and decrease their children’s quality of life.

## Method

### The study’s goal, design, and setting

The primary objective of this research was to investigate how mothers of children with cancer perceive ambiguous bodily information in their kids, comparing their interpretations to those made by mothers of children without cancer. In addition, we were interested to know more about the mediating role of HA in the relationship between negative interpretation bias (IB) and FoP.

### Participants

Both the mothers of children with cancer and their kids, and the mothers of children without cancer and their kids (control group) were recruited in this study. The mothers and children with cancer were recruited from a children’s hospital (Ali Asghar Hospital, Tehran, Iran), where a temporary experimental psychology testing setup had been established. All the kids were undergoing active cancer treatment for leukemia. Also, the mothers of children and their kids who did not have cancer were recruited from social meeting centers by placing advertisements in public places. The inclusion criteria for all participants required them to be native Farsi speakers. Mothers in the cancer group were required to have children between the ages of 7 and 16 diagnosed with leukemia and currently undergoing treatment, and mothers in the control group were required to have children of the same ages who had no history of serious illnesses. All mothers needed to have normal or corrected-to-normal vision and literacy skills to complete the task. Exclusion criteria included a history of psychiatric disease and being under medication or unable to comply with the task requirement (putting noise-canceling headphones, being in a separate room, concentrating on the task). A total of 3 mothers (2 in the cancer group and one in the control group) were excluded because they did not complete the task and decided to leave before the end of the test session. We adhered to the standards specified according to the Declaration of Helsinki. All participants received the consent form and approved their participation before the beginning of the session. The ethics committee of the Institute for Cognitive Science Studies approved the study.

### Interpretation bias task

The IB task’s aim was to examine systematic bias in the processing of ambiguous information. We developed and presented the task in PsychoPy3 software (Nottingham, UK). Participants completed a computerized IB assessment task. A fixed dot appeared on the center of the monitor for 500 ms at first. Then, one by one, three sentences appeared on the screen. Each sentence took 1000 ms to read; for instance: Your child is playing in the snow/suddenly slips/and she falls to the ground with her hands. The screen then displays the fourth sentence, which has a blank word (for example, and she breaks her ----). Then, in the center of the screen, three words that could be regarded as negative (e.g., wrist), positive (e.g., wristwatch), or neutral (e.g., book) were displayed. By selecting the appropriate button on the response box, the participant was required to indicate which word came to their mind immediately or was closest to the first term. The subsequent trial started immediately after the participant’s response. There were 32 situational scenarios, 16 ambiguous, 8 health-related, and 8 non-health-related scenarios. Before the main block, there were two additional training trials. The rate of negative, positive, or neutral selected statements was calculated for each participant. A negative interpretation trial is one in which the participant responded to an ambiguous scenario in a health-threatening way, and a positive interpretation trial means the participant responded to an ambiguous scenario in a non-health-threatening way.

The validity of our IB task is supported by previous research that has employed similar tasks to assess IB in various contexts. The use of ambiguous scenarios followed by word choices with different valences (negative, positive, or neutral) has been shown to be effective in capturing biased interpretations in individuals [29, 30]. Additionally, the reliability of these tasks has been tested in numerous studies in order to distinguish between individuals with and without anxiety [29, 31]. Also, several studies found that using computerized tasks to assess IB is more accurate and consistent than other methods (e.g., self-report) [32].

### Health Anxiety Inventory-Short Form (HAI-18)

The Short Health Anxiety Inventory (HAI) is a self-report questionnaire with 18 items that assess physical health–related anxiety [33]. Each item consists of four statements referring to a certain aspect of health concerns. The total score can range from 0 to 54. It has been demonstrated that the Farsi version of the questionnaire is a valid and reliable measurement tool by its usage in some previous studies (Cronbach’s alpha = 0.75) [34].

### Fear of Progression Questionnaire-Short Form-Parent version (FoP-Q-SF/PR)

This questionnaire consists of four parts (affective reactions, partnership/family, occupation, and loss of autonomy) with 12 items ranked on a 5-point Likert scale (never=1, very often=5; total score ranged between 12 and 60). The internal consistency of the FoP-Q-SF/PR in parents of children with cancer was good, with Cronbach’s alpha = 0.89 [35]. This measure has been translated to Farsi, validated in Iran, and demonstrated to be a reliable and valid measure (Cronbach’s alpha total score = 0.77).

### Health-related quality of life (HRQoL)-Parent proxy and Child version-Short Form (Kidscreen-27)

This questionnaire has five parts and 27 questions; these five parts are based on the Rush scale: physical well-being (5 questions), psychological well-being (7 questions), autonomy and parent relation (7 questions), social support and peers (4 questions), and school (4 questions). Cronbach’s alpha of all five parts is above 0.7 [36]. The Farsi version of the questionnaire has been used in some prior studies and demonstrated to be a valid and reliable measurement instrument [37]. In the present study, Cronbach’s alpha for parent proxy and child versions was 0.85 and 0.87, respectively.

### Procedure

The study was conducted in an isolated room at Ali Asghar Hospital in Tehran, Iran. The experimenter welcomed the participants and guided them to the room. Only the participant and the experimenter were present in the room. Participants first read and signed the consent form. Then, they were given instructions about the task and had the opportunity to complete two trials while the experimenter was in the room. Then, they had to put the noise-canceling headphone on, and the experimenter left the room to avoid disturbing the participant. After completing the task, the participant was asked to complete the questionnaires online in the same session using the same laptop. In the end, the participant was debriefed, and the session ended.

### Statistical analysis

**Fig. 1:**
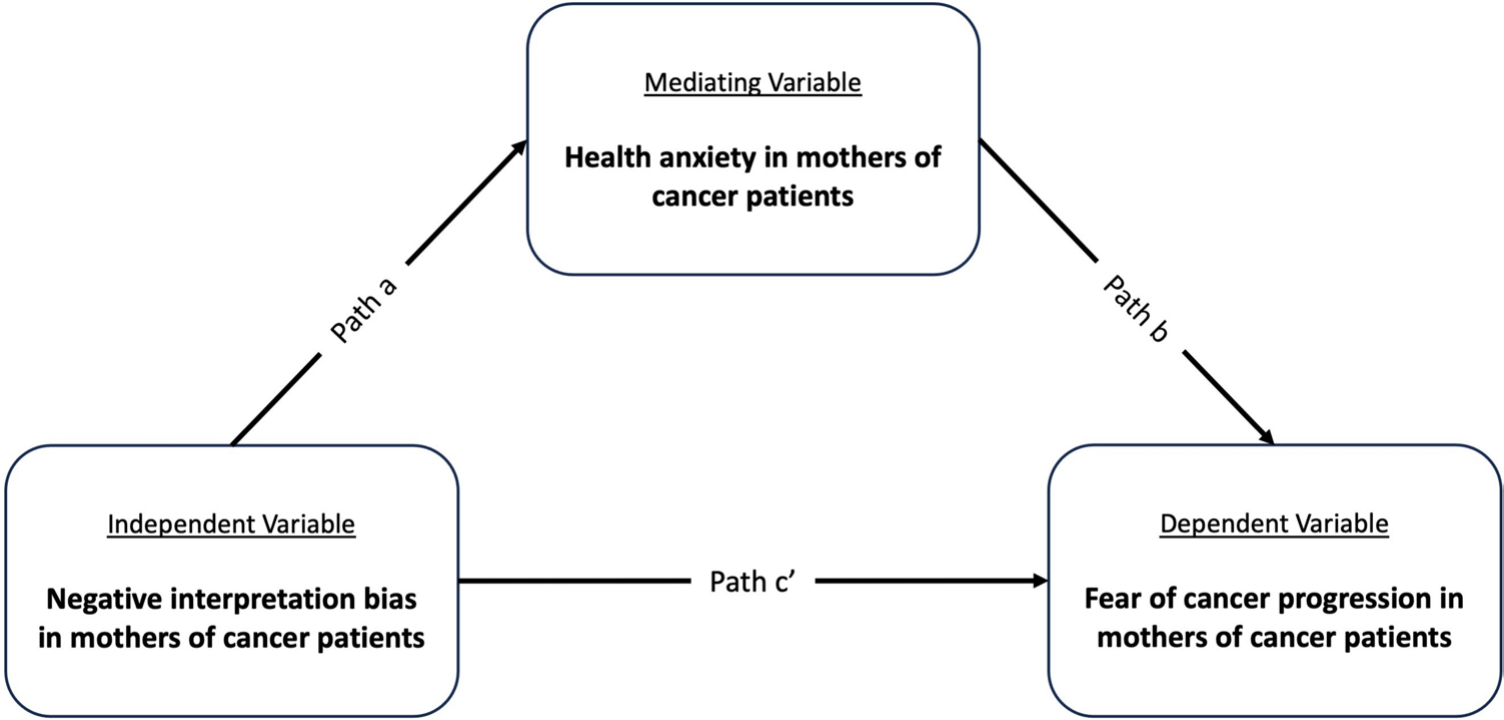
The relationship between negative interpretation bias and fear of cancer progression in mothers of patients with cancer is mediated by their health anxiety

Statistical significance was set at a level of <0.05 for all analyses. Based on the hypothesis and our previous study [28], we calculated the sufficient sample size to have statistical power for the analysis of our data. The HAI and FoP-Q-SF/PR total scores were calculated according to the instructions. In addition, a negative IB score was determined by counting the number of negative responses selected by the participant. Statistical packages in R-Studio were used to report descriptive statistics, such as the basic information about the sample, the mean, and the standard deviation. We investigate the relationship between IB, HAI, and FoP-Q-SF/PR, to examine the mediating effect of HA on the relationship between a negative IB (independent variable) and FoP (dependent variable) (Fig. 1). For this analysis, the SPSS “PROCESS” macro model 4 was used [38].

## Results

### Descriptive statistics

In the present study, 114 mothers were invited to participate. After applying the exclusion criteria, 3 participants were excluded. The final sample consisted of 111 mothers, with 69 mothers having children currently undergoing cancer treatment and 42 mothers having healthy children. The participants’ demographic information, including ages, sex, marital status, and education, are presented in Table 1. As demonstrated in Table 1, there was no difference between mothers of cancer patients and the control group regarding the age and sex of children. Still, there was a difference in marital status and level of education between the two groups. The fact that both the skewness and kurtosis indices fall within the ±1.96 range provides further evidence that the data follow a normal distribution.

**Table 1:** Descriptive information (age, gender, marital status, education)s

	Group		*t*/*x*^2^	*df*	*p*-value
	Patient*	Control			
Number of participants	69	42			
Age (mean ± SD)					
Mother age	37.43±4.69	39.21±4.90	18.027	20	0.586
Child age	9.88±2.74	10.54±2.71	5.934	10	0.821
Sex of children			1.830	1	0.176
Male	32	14			
Female	37	28			
Marital status			6.248	1	0.012
Single	7	12			
Married	62	30			
Education			11.522	2	0.003
Secondary school and lower	28	5			
Diploma	35	28			
Bachelor and upper	6	9			

*Patient: This column refers to the mothers of a child with cancer

As shown in Table 2, using the independent samples, a *t*-test showed that mothers of cancer patients had more negative IB and higher HA than in the control group. In addition, it showed that positive IB and HRQoL in parent proxy and child versions in mothers of cancer patients were much less than in the control group (*p*s <0.001).

**Table 2:** Mothers of cancer patients and control groups were compared on negative interpretation bias, positive interpretation bias, health anxiety, health-related quality of life, and fear of progression (mean ± SD)

	Patient*	Control	*t*	*p*-value
Negative interpretation bias	10.16±2.09	6.19±2.09	9.703	< 0.001
Positive interpretation bias	5.79±2.05	9.81±2.09	−9.918	< 0.001
Health anxiety	31.36±6.41	14.95±4.03	14.89	< 0.001
Health-related quality of life parent proxy	75.38±8.59	100±8.85	−14.62	< 0.001
Health-related quality of life for children	74.85±9.43	96.76±10.73	−11.26	< 0.001
Fear of progression	33.58±4.92	_		

*Patient: This column refers to the mothers of a child with cancer

The correlation analysis revealed a strong positive association between the negative IB and HA (*r* (68) = 0.406, *p* < 0.001). It means increasing HA in mothers of cancer patients was related to more negative choices in ambiguous scenarios. In addition, HA was correlated with FoP positively (*r* (69) = 0.467, *p* < 0.001), implying that the more mothers of cancer patients worry about the progress of their child’s cancer, the more anxious they are. Moreover, negative IB is positively correlated with FoP (*r* (68) = 0.525, *p* < 0.001), indicating that when mothers of cancer patients choose more negative interpretations in ambiguous scenarios, it is associated with increased fear of the progress of their child’s cancer. Also, there is a significant negative correlation between FoP and HRQoL in patients’ mothers (*r* (69) = −0.390, *p* < 0.001), suggesting that increased FoP in mothers of cancer patients is associated with decreased HRQoL from mothers’ view towards their children. Finally, a positive correlation has been observed between HRQoL scores in mothers of children with cancer and HRQoL scores in their children (*r* (69) = 0.602, *p* < 0.001). This suggests that a decline in the quality of life experienced by these mothers might be associated with a reduced quality of life in their children.

### Mediation analysis

Lastly, mediating analysis (model 4) by using PROCESS for SPSS was used to examine the effect of HA as a mediator between negative IB and FoP.

Our analysis showed that the total effect of negative IB on FoP in mothers of cancer patients was significant (*r* = 0.525, *p* < 0.001, *r*^2^ = 0.275). The direct effect of negative IB on mothers’ FoP was significant (path *c*′, *b* = 0.950, se = 0.259, *p* < 0.001), suggesting that a more negative interpretation of ambiguity was associated with higher FoP. Also, the direct effect of HA on FoP was positive and significant (path *b*, *b* = 0.233, se = 0.084, *p* = 0.007), suggesting that higher HA in mothers contributes to higher FoP in them. Furthermore, the indirect effect of negative interpretations on mothers’ FoP through mediating role of HA was significant (path a*b, IE=0.294, 95% CI=0.202–0.6876).

## Discussion

In this research, we investigated how mothers of cancer patients interpreted ambiguous health information in a biased manner. We also examined the relationship between IB among mothers of children with cancer and FoP in their children and investigated the mediating role of mothers’ HA in this relationship. Our findings indicated that mothers of kids with cancer interpret ambiguous health-related information more negatively than mothers of kids without cancer as the control group. Moreover, we observed a positive correlation between negative IB and FoP among mothers of cancer patients. It suggests a more negative interpretation of health-related ambiguity among mothers of kids with cancer is associated with higher fear related to the expectation of cancer progression. Moreover, there was a significant negative correlation between FoP and HRQoL among mothers of kids with cancer, which means increased FoP was associated with decreased kids’ HRQoL from mothers’ views. Finally, HRQoL in mothers of cancer patients was positively associated with HRQoL in their children, indicating that lower quality of life in mothers with cancer kids is associated with lower quality of life in their children. Finally, among mothers of kids with cancer, HA played a mediating role in the association between IB and FoP. This mediation suggests that more negative IB can increase mothers’ HA. Increased HA can contribute to elevated levels of fear of kids’ cancer progression among mothers.

Cancer does not always recur, but like many other chronic conditions, its experience may impact patients and their families, psychologically or financially. Many patients deal with the fear of recurrence or progression of their disease. FoP is a normal and appropriate reaction to the real threat of cancer [39]. High levels of FoP, on the other hand, can become dysfunctional, hurting well-being, quality of life, and social interaction [40]. According to previous study, one of the most common distressing symptoms of cancer patients is FoP [23]. Also, FoP has been found to play a significant role in the quality of life of a patient with chronic disease [41]. Many studies demonstrated that patients’ biased interpretation of disease-specific information is associated with their FoP [28, 42, 43]. For instance, a recent study demonstrated that women with cancer interpreted ambiguous words as more health-threatening than individuals without cancer [44]. This bias was more significant for women with higher FoP. A recent study revealed that women with breast cancer tend to interpret ambiguous scenarios with cancer-related words, which was associated with their fear of recurrence [45]. We found that negative interpretation in cancer patients’ mothers has a positive association with their FoP of kids’ cancer.

Fear of progression can impact patients’ and caregivers’ quality of life [46]. Because of the caregiving burden, the quality of life of cancer patients’ caregivers is susceptible to decline [47]. Moreover, higher cancer severity was associated with higher levels of fear of recurrence among survivors and caregivers, associated with a poorer quality of life [48]. In addition, mothers with children diagnosed with cancer rated their own and their child’s quality of life significantly lower than the average population; also, there was a significant association between the mother’s rating of their quality of life and the child’s quality of life [49]. Similarly, we found that in mothers of cancer patients, there is a negative relationship between FoP and quality of life towards their kids, and also between the quality of life of children from the point of view of mothers and what the children themselves rate their quality of life is a correlation. It means when FoP in mothers increases, their own and their children’s quality of life decreases, thus more negative interpretations

Health anxiety is an obsession with having or contracting a significant illness and can impact patients’ quality of life [50]. People with HA overcheck their bodies for signs of disease, leading to an increased referral to hospitals or complete avoidance to prevent anxiety related to the expectation of test results [51]. Several studies in different populations suggest increased HA among patients suffering from chronic disease [52, 53]. Some of these studies also have shown that for the conditions with a chance of relapse, HA contributes to fear related to relapse among the patients [54]. Our findings take one step further and suggest that a caregiver’s anxiety about the health status of the patients can impact caregivers’ fear of the progression of the patient’s diseases.

Based on recent studies on patients with chronic disease, fear management plays a critical role in enhancing patients’ quality of life [55]. In some therapeutic populations, it is not feasible to target fear directly since exposure to fear may elicit unpredictable responses [56]. Thus, changing HA can indirectly reduce the FoP and improve the quality of life among patients and caregivers.

Understanding the factors that impact HA may play an essential role in improving a patient’s quality of life. The cognitive-behavioral model of HA highlights factors contributing to and maintaining HA. Cognitive aspects include overestimating the risk of having or developing a serious illness, overestimating its seriousness, and misinterpreting body sensations/functions/appearance as signs of serious illness [57]. This model of HA considers threat interpretations as fundamental to the anxiety experience. These interpretations lead to the feeling of anxiety, bodily response, and reassurance behaviors, which all contribute to health appraisals. According to this model, greater negative interpretation of ambiguous information contributes to increased levels of HA in patients. In fact, and in line with other studies, people with chronic diseases with higher HA tend to fear more about having a relapse [28]. Furthermore, HA is a mediating component between IB and fear of relapse among other chronic patients. For instance, the level of HA in RRMS patients is substantially higher than in matched participants without the condition [28, 54]. Similar results were obtained in this research, so the increase in HA in mothers was related to the increase in the FoP towards their kids.

Among patients with chronic diseases, cognitive bias plays a critical role in the onset and maintenance of psychological difficulties [58]. For example, studies have shown that people with chronic pain are more likely to interpret ambiguous pain-related information more negatively than people without pain [59]. Also, caregivers of patients with chronic disorders tend to have cognitive biases like their patients and develop psychological issues [17, 60]. Previous research has shown that mothers of patients with chronic pain interpret ambiguous pain-related information negatively from pain-free people [16]. Similar to previous theories, our findings supported our hypothesis that mothers of cancer patients have negative IB for ambiguous bodily information towards their kids. This negative bias can be considered a protection strategy that may increase survival chance: if they misinterpret ambiguous as negative, consequences are less than if they misinterpret signs related to progression as benign [61]. However, in chronic conditions, this negative interpretation can contribute to FoP, as suggested by our findings.

Interpretation bias modification (IBM) has been suggested to effectively change negative IB and reduce anxiety [32, 62]. Incorporating IBM in the management of HA related to negative IB could offer potential benefits for mothers of cancer patients. IBM focuses on training individuals to generate more benign interpretations of ambiguous information by encouraging the participant to resolve the ambiguity in a non-threatening way [32]. By consistently practicing the generation of non-threatening interpretations, individuals can gradually shift their IB towards a more balanced perspective. Therefore, changing negative IB to positive or benign interpretations can be considered an intervention to reduce HA, as indicated by previous studies [63]. However, it is essential to consider the limitations and challenges associated with implementing this technique, such as individual differences in response to intervention and the need for further research to optimize its efficacy.

Despite its originality and significance, this study has some limitations that should be considered when interpreting the results. Because of the COVID-19 pandemic and quarantine, we could not recruit more people in public places, so the number of people in the control group was less than in the clinical group. Also, the hospital did not allow us to access the children’s files to know the stage of their cancer, which could affect the results [64]. Furthermore, because of the restricted access to the hospital for non-patients, the clinical sample and the control group were separately recruited. The context may influence respondents’ answers, and in future studies, recruiting and testing participants in the same context are suggested.

## Conclusion

Here we showed that kids with cancer and their mothers have a lower quality of life than those without a cancer history. We suggested that a higher HA and FoP reduce the patient’s and the mother’s quality of life. We found that negative IB in mothers of cancer patients contributes to increased HA, which indirectly increases the FoP of their kids’ cancer and decreases the mother’s and child’s quality of life. Moreover, given the evident role of negative IB in contributing to increased HA and FoP, it seems worthwhile to consider intervention strategies such as IBM. IBM, which involves training individuals to generate more benign interpretations of ambiguous information, could offer potential benefits in managing HA associated with negative IB, particularly for mothers of children with cancer. By changing negative IB, IBM may help to reduce HA and FoP, thereby potentially improving the quality of life for both mothers and their children. Therefore, considering both our findings and the potential benefits of IBM, we propose that changing HA through modification of IB might lower the FoP in mothers of kids with cancer and improve the mother’s and child’s quality of life.

## Data Availability

The dataset can be obtained by contacting the corresponding author.
